# Cell Recovery in Bronchoalveolar Lavage Fluid in Smokers Is Dependent on Cumulative Smoking History

**DOI:** 10.1371/journal.pone.0034232

**Published:** 2012-03-29

**Authors:** Reza Karimi, Göran Tornling, Johan Grunewald, Anders Eklund, C. Magnus Sköld

**Affiliations:** Division of Respiratory Medicine, Department of Medicine, Karolinska Institutet, Karolinska University Hospital Solna, Stockholm, Sweden; University Hospital Freiburg, Germany

## Abstract

**Background:**

Smoking is a risk factor for various lung diseases in which BAL may be used as a part of a clinical investigation. Interpretation of BAL fluid cellularity is however difficult due to high variability, in particular among smokers. In this study we aimed to evaluate the effect of smoking on BAL cellular components in asymptomatic smokers. The effects of smoking cessation, age and gender were also investigated in groups of smokers and exsmokers.

**Methods:**

We performed a retrospective review of BAL findings, to our knowledge the largest single center investigation, in our department from 1999 to 2009. One hundred thirty two current smokers (48 males and 84 females) and 44 ex-smokers (16 males and 28 females) were included. A group of 295 (132 males and 163 females) never-smokers served as reference.

**Result:**

The median [5–95 pctl] total number of cells and cell concentration in current smokers were 63.4 [28.6–132.1]×10^6^ and 382.1 [189.7–864.3]×10^6^/L respectively and correlated positively to the cumulative smoking history. Macrophages were the predominant cell type (96.7% [90.4–99.0]) followed by lymphocytes (2% [0.8–7.7]) and neutrophils (0.6% [0–2.9]). The concentration of all inflammatory cells was increased in smokers compared to never smokers and ex-smokers. BAL fluid recovery was negatively correlated with age (p<0.001). Smoking men had a lower BAL fluid recovery than smoking women.

**Conclusion:**

Smoking has a profound effect on BAL fluid cellularity, which is dependent on smoking history. Our results performed on a large group of current smokers and ex-smokers in a well standardized way, can contribute to better interpretation of BAL fluid cellularity in clinical context.

## Introduction

Bronchoalveolar lavage is a noninvasive method which allows sampling of cells and soluble components from the lower respiratory tract [1], [2], [3], [4], [5]. The method was introduced in the 1970s [6] and has gained acceptance as a research but also as a diagnostic tool. The differential cell count in BAL fluid may provide information supporting the diagnosis of diffuse interstitial lung diseases. For instance, in idiopathic pulmonary fibrosis neutrophils and eosinophils are increased, and in hypersensitivity pneumonitis and sarcoidosis a lymphocytic alveolitis is seen [7], [8], [9], [10]. Cellular analyses of BAL fluid in combination with clinical and chest radiographic findings may thus reduce the need for more invasive biopsy procedures. Furthermore, in a few rare diseases such as pulmonary Langerhans cell histiocytosis with increased proportion of CD1a+ cells and idiopathic pulmonary hemosiderosis with hemosiderin laden macrophages, BAL can confirm the diagnosis [9], [11]. Since BAL samples the distal part of the lung, i.e. the alveoli and the small airways, and the epithelial lining fluid is directly exposed to the environment, the exposure situation and the local milieu have a substantial impact both on the cellular and non-cellular components of the recovered fluid [9], [12], [13], [14], [15], [16]. Cigarette smoking is a well-defined common pollutant, which influences both cellular and soluble components of BAL fluid. For example, smoking subjects have a significant elevated number of cells in the lower respiratory tract, mainly due to an increased number of alveolar macrophages [8], [14], [17], [18]

Cigarette smoking is also a risk factor for various lung diseases [19], [20], [21], [22] such as chronic obstructive pulmonary disease (COPD), usual interstitial pneumonia (UIP), desquamative interstitial pneumonia (DIP), respiratory bronchiolitis associated interstitial lung disease (RB-ILD) and pulmonary Langerhans cell histiocytosis (PLCH). Since these patients may undergo BAL as a part of a clinical investigation [19], [23], it is important to distinguish inflammatory changes due to cigarette smoking *per se* from changes due to the disease. The establishment of a standardized reference material for BAL in asymptomatic smokers may contribute to a better interpretation and utility of this important diagnostic tool in a clinical framework.

In this retrospective analysis we therefore investigated effects of cigarette smoking on BAL fluid cellular findings in a large number of healthy asymptomatic smokers and ex-smokers with the aim of establishing reference values to be used in a clinical setting. Specifically, we made an effort to elucidate the effects of accumulated smoking history, age and gender on BAL fluid cell contents. In addition, the long term effect of smoking cessation was addressed.

## Materials and Methods

### Study design

We performed a retrospective analysis of BAL investigations done at our department on subjects who had participated as healthy volunteers in various research projects from 1990 to 2009. All individuals were recruited by word of mouth or by advertisement in local newspapers and were reimbursed for their participation.

### Ethics statement

This was a retrospective study and all the subjects had previously participated as control groups in different studies, conducted during 1990–2009. All these individual studies had previously been approved by the Regional Ethical Review Board in Stockholm, Sweden. Informed consent has been obtained previous from all participants in each study. In the 90s only verbal consent was required. In studies performed in the 2000s also written consent has been obtained. In the present retrospective study, all data were analyzed anonymously.

### Subjects

We identified 132 subjects (48 males and 84 females) who were current smokers at the time of bronchoscopy. Their smoking history was recorded and is presented as pack-years (Table 1). Current smokers older than forty years of age underwent a dynamic spirometry (Vitalograph MDI-Compact; Buckingham Hamburg, Germany), and they were included only if they had a FEV_1_/FVC>0.7 and a FEV_1_>80% of predicted normal values according to the European Community for Steel and Coal (ECSC) [24]. Subjects with allergy, asthma or any clinical history of other respiratory diseases were excluded. All subjects underwent a posterior-anterior and lateral chest X-ray and a routine medical examination, and all findings had to be within the normal range. No clinical signs of upper or lower respiratory infection for at least four weeks before investigation were allowed. We also included 44 ex-smokers (16 males 28 females) who had quit smoking at least ten months prior to the investigation and fulfilled the same inclusion and exclusion criteria as the current smokers. As a nonsmoking reference group we used a cohort of 295 never smokers (163 females and 132 males) who are described in detail elsewhere (Olsen HH et al, submitted).

**Table 1:** Characteristics of the study subjects.*

	Current Smokers(N = 132)	Ex-Smokers (N = 44)	Never Smokers (N = 292)
Age	39.3±13.9 (20–66)	38.6±8.0 (26–54)	31.5±11.7 (18–65)^b^^,^ c
Males/females	48/84	16/28	132/163
Pack-years	20.8±15.1(2–84)^a^	5.3±6.7(0.2–35)	N.A.
Time since smoking cessation (months)	N.A.	117.6±77.3 (10–336)	N.A.

* Values are expressed as mean±SD and range within brackets.

a p<0.0001 “Current Smokers” vs. “Ex-smokers”.

b p<0.0001 “Current Smokers” vs. “Never Smokers”.

c p<0.0001 “Ex-smokers” vs. “Never Smokers”.

N.A. not applicable.

### Bronchoscopy and BAL

The subjects were fasting for at least eight hours prior to the procedure. Participants received pre-medication with morphine-hyoscin or pethidine and atropine intramuscularly 45 minutes before the investigation. Bronchoscopy and BAL was then performed in the morning by experienced bronchoscopists assisted by research nurses according to a standardized protocol on an outpatient basis. All bronchoscopies were performed in our department. Briefly, after topical anesthesia with lignocaine, the bronchoscope (Olympus F Type P 30 or equivalent instruments; Olympus Optical Co. Ltd, Tokyo, Japan) was inserted nasally with the subject in supine position. The tip of the bronchoscope was wedged in a subsegmental bronchus of the middle lob, or in a few cases in the lingula lobe. Five aliquots of 50 mL phosphate-buffered saline solution at 37°C were instilled. After each instillation, the fluid was gently suctioned back with a negative pressure of −40 to −50 mm Hg. If the recovery appeared to be poor, the suction pressure was occasionally adjusted to −10 to −20 mmHg. Dwell time was kept to a minimum as recommended by the European Respiratory Society (ERS) task force [25]. The five BAL fluid aliquots were pooled and collected in a silicone treated plastic bottle which was kept on ice and immediately transported to the laboratory.

### Preparation of BAL fluid

All BAL fluids were prepared and analyzed at the Lung Research Laboratory at the Department of Medicine at Karolinska Institutet by experienced laboratory technicians. The BAL fluid was filtered through a Dacrone layer (Type AP32; Millipore, Cork, Ireland). The volume of the recovered fluid was measured and the recovery percentage was calculated. Cell pellet was prepared by centrifugation at 400×g for ten minutes at 4°C, and was then re-suspended in RPMI 1640 medium (Sigma, St. Louis, USA). Cell count and cell viability were assessed after staining with trypan blue, using a Bürker Chamber (Marienfeld, Germany). Cytocentrifugation (Cytospin 2; Shandon LTD, Runcorn, UK) at 22×g for three minutes was employed for cell differential count. After staining with May-Grünwald Giemsa 500 cells were counted. Mast cells were stained with toluidine blue, and the number of cells within 10 visual fields (16×magnifications) was scored and reported as absolute number of these cells. Findings were reported as both total cell count and cell concentration, and differential cell count as concentration and percentage of the total cell number.

### Statistical analysis

Descriptive statistics were used to define the reference values defined as the 5^th^ and 95^th^ percentiles for current smoking subjects. Comparisons between groups were performed by analysis of variance, using the Satterthwaite approximation in case of unequal variance between the groups. In order to examine the influence of smoking history (pack years) and age on BAL fluid components we employed a stepwise regression analysis using Pearson correlation coefficient. Since the study was regarded as exploratory, no corrections due to multiple analyses were performed in order to avoid false negative conclusions and a p value<0.05 was considered significant. However, p-values above 0.005 should be interpreted with caution.

## Results

### Recovery, total cell count and differential cell count in current smokers

Results from the current smoking group are presented in Table 2. Recovery of instilled fluid ranged from 34% to 82%, and the median viability was 91% (range 70% to 100%). The median total number of cells and cell concentration were 63.4×10^6^ and 382.1×10^6^/L respectively. The inter-individual variability was large ranging from 11.5×10^6^ to 177.8×10^6^ for total cell count and 67.5×10^6^/L to 1280×10^6^/L for cell concentration respectively, and the variability showed a tendency to be more pronounced at higher ages. The majority of cells were alveolar macrophages (median 96.7%; range 73.2–99.6%) followed by lymphocytes (median 2%; range 0.2–26%) and neutrophils (median 0.6%; range 0–6%). Basophils, eosinophils and mast cells were represented scarcely. As for total cell number and cell concentration, the inter-individual variability was large for the differential counts, in particular with regard to the concentration of macrophages which varied by a factor 20 between minimum and maximum (64.1×10^6^/L and 1274×10^6^/L respectively).

**Table 2:** Descriptive statistics of BAL findings in current smoking subjects.

Variable	N	Minimum	Median	Maximum	5^th^ Pctl	95^th^ Pctl
Return volume (mL)	128	86	164	204	119	188
Recovery (%)	128	34	66	82	48	75
Viability (%)	132	70	91	100	80	99
Total cell number (10^6^)	128	11.5	63.4	177.8	28.6	132.1
Cell concentration (10^6^/L)	128	67.5	382.1	1280	189.7	864.3
Macrophages (%)	132	73.2	96.7	99.6	90.4	99.0
Macrophages (10^6^/L)	128	64.1	356.8	1 274.9	172.2	842.2
Lymphocytes (%)	132	0.2	2	26	0.8	7.7
Lymphocytes (10^6^/L)	128	1.1	7.8	65.7	2.2	34.8
Neutrophils (%)	131	0	0.6	6	0	2.9
Neutrophils (10^6^/L)	127	0	2.5	30.4	0	13.2
Eosinophils (%)	132	0	0	3.6	0	1.4
Eosinophils (10^6^/L)	128	0	0	14.6	0	5.5
Basophils (%)	132	0	0	2	0	0.1
Basophils (10^6^/L)	128	0	0	4.4	0	0. 5
Mast cells (per 10 visual fields)	65	0	2	13	0	8.8

### Correlations with smoking history and age

**Figure 1:**
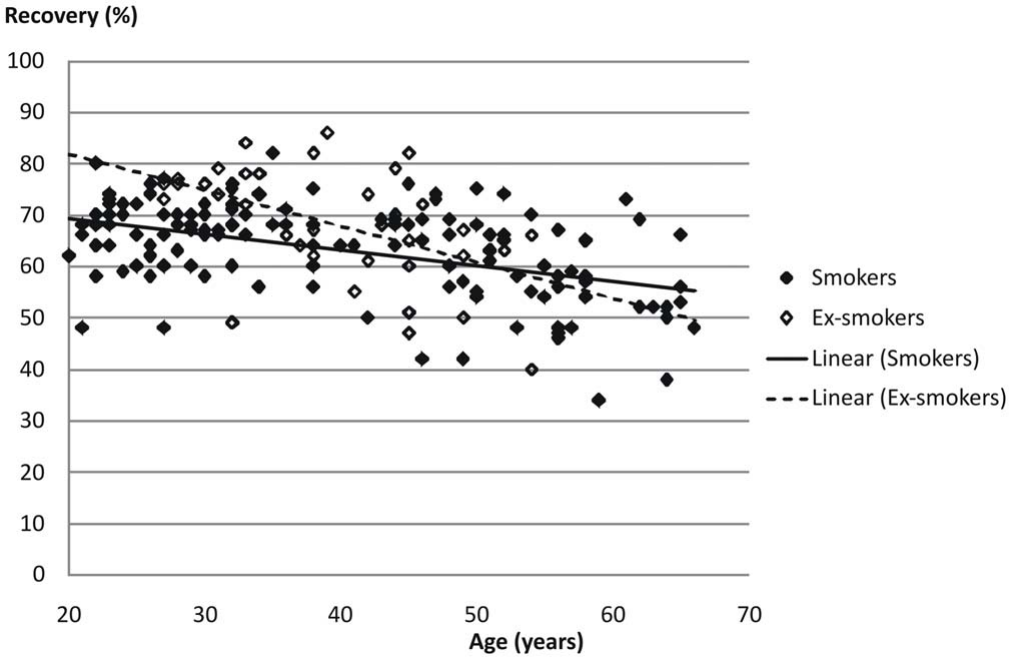
Relation between percentage of recovered fluid in BAL and age from current smokers and ex-smokers.

**Figure 2:**
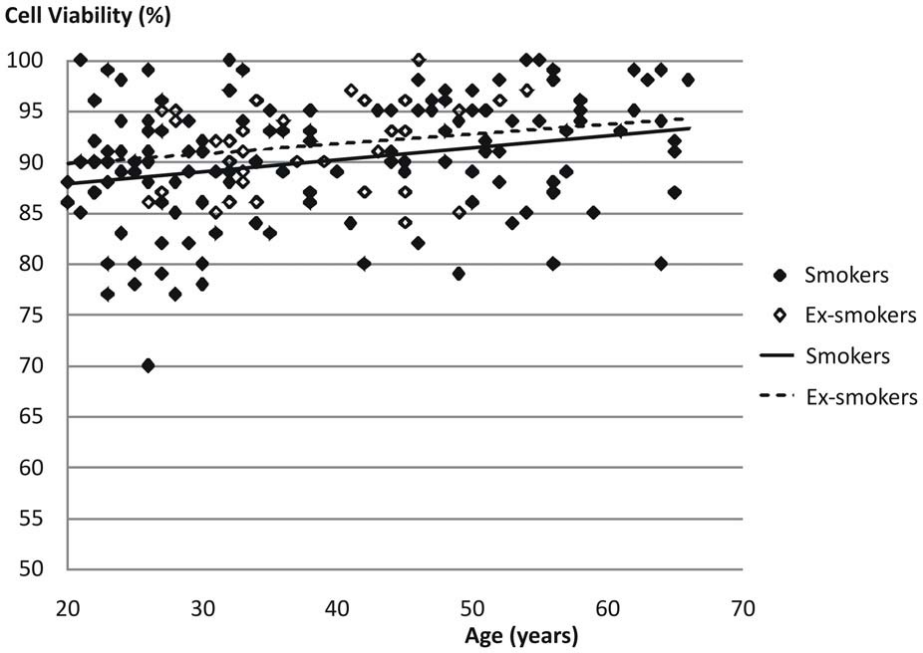
Relation between cell viability in BAL fluid and age from current smokers and ex-smokers.

**Figure 3:**
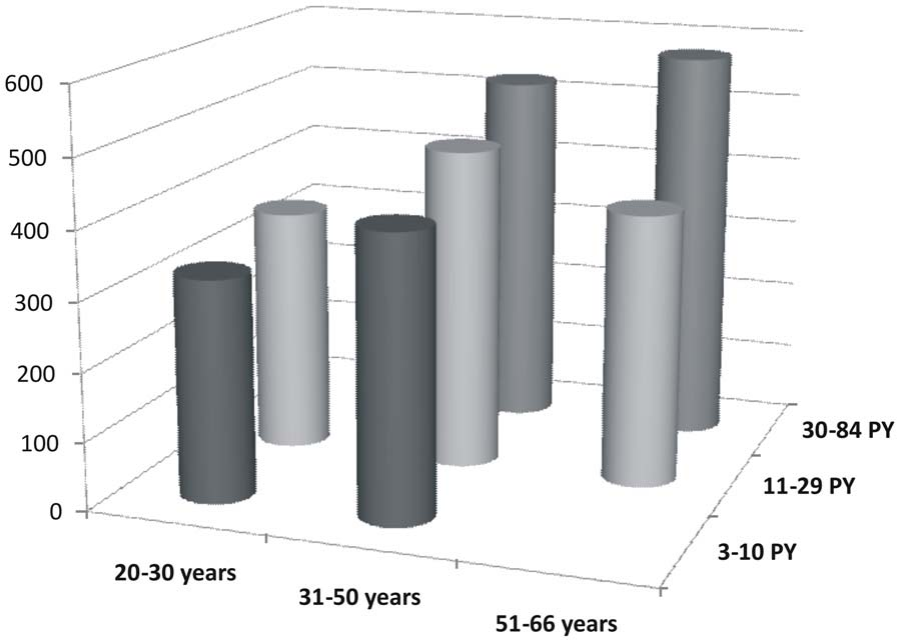
Total cell concentration (10^6^/L) in relation to age and smoking history in BAL fluid from current smokers, in three different groups according to age and smoking history expressed as pack-years (PY).

**Figure 4:**
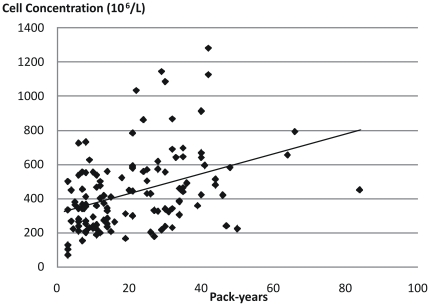
Relation between cell concentration (10^6^/L) in BAL fluid and smoking history expressed in pack-years, from current smokers.

**Figure 5:**
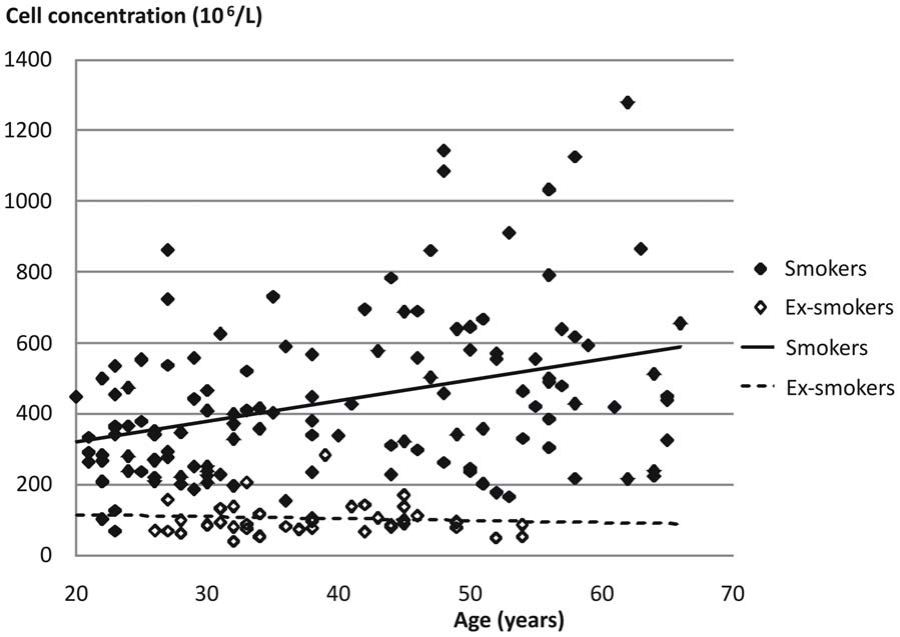
Relation between cell concentration and age in BAL fluid from current smokers and ex-smokers.

Both return volume and percentage of recovered fluid showed a statistically significant correlation with age (p<0.001 for both) but not with smoking history (Table 3 and Figure 1). Cell viability was negatively correlated with age (p<0.01) but no influence could be seen from smoking history (Figure 2). Cell concentration was significantly (p<0.001) correlated with smoking history but not with age (Table 3, Figure 3, Figure 4 and Figure 5). As for total cell number and cell concentration, there was a statistically significant (p<0.001) correlation between macrophage concentration and cumulative smoking history, but there was no correlation with age. The percentage of eosinophils showed a statistically significant correlation (p<0.05) with age but not with smoking history.

**Table 3:** Step wise regression of pack-years and age with BAL findings in current smoking subjects.

	Model	Intecept		Pack-years		Age	
Variable	p	Estim. (SE)	p	Estim. (SE)	p	Estim.(SE)	p
Return volume (mL)	<.001	187.8 (5.6)	<.001	…	n.s.	−0.7 (0.1)	<.001
Recovery (%)	<.001	75.1 (2.2)	<.001	…	n.s.	−0.3 (0.1)	<.001
Viability (%)	<.01	85.8 (1.5)	<.001	…	n.s.	0.1 (0.0)	<.01
Total cell number (10^6^)	<.01	55.5 (5.2)	<.001	0.6 (0.2)	<.01	…	n.s.
Cell concentration (10^6^/L)	<.001	313.3 (32.8)	<.001	5.8 (1.3)	<.001	…	n.s.
Macrophages (%)	n.s.	…	n.s.	…	n.s.	…	n.s.
Macrophages (10^6^/L)	<.001	297.7 (31.8)	<.001	5.7 (1.2)	<.001	…	n.s.
Lymphocytes (%)	n.s.	…	n.s.		n.s.	…	n.s.
Lymphocytes (10^6^/L)	n.s.	…	n.s.	…	n.s.	…	n.s.
Neutrophils (%)	n.s.	…	n.s.	…	n.s.	…	n.s.
Neutrophils (10^6^/L)	n.s.	…	n.s.	…	n.s.	…	n.s.
Eosinophils (%)	<.05	.628 (.145)	<.001	…	n.s.	.001 (.003)	<.05
Eosinophils (10^6^/L)	n.s.	…	n.s.	…	n.s.	…	n.s.
Basophils (%)	n.s.	…	n.s.	…	n.s.	…	n.s.
Basophils (10^6^/L)	n.s.	…	n.s.	…	n.s.	…	n.s.
Mast cells (per 10 visual fields)	n.s.	…	n.s.	…	n.s.	…	n.s.

### Comparison between smokers, ex-smokers and never smokers

**Figure 6:**
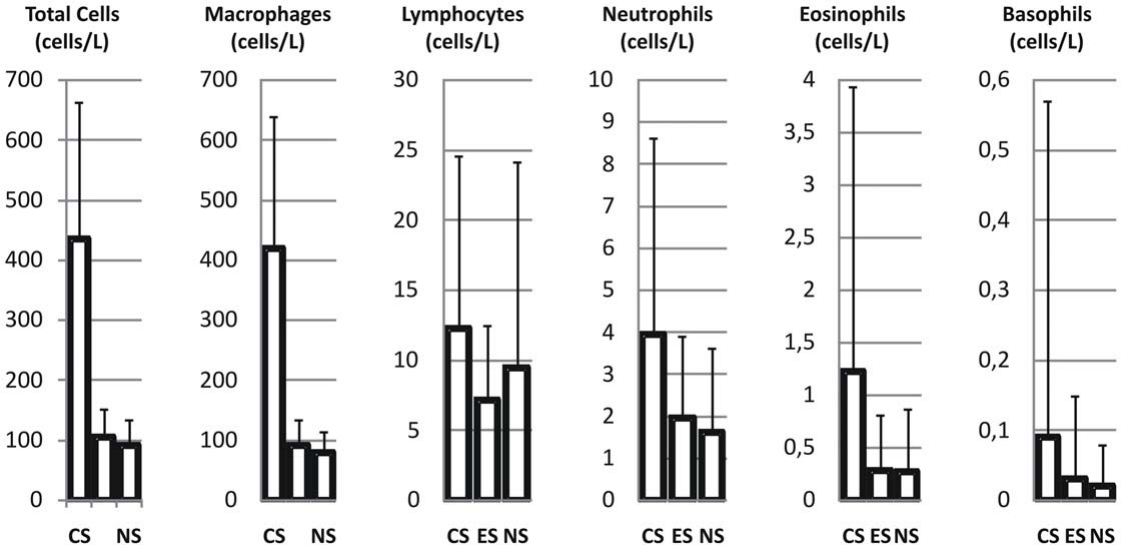
Cell concentration (10^6^/L) of the various inflammatory cells in BAL fluid in current- smokers (CS) and never-smokers (NS) and ex-smokers (ES). Mean and standard deviation are given.

**Figure 7:**
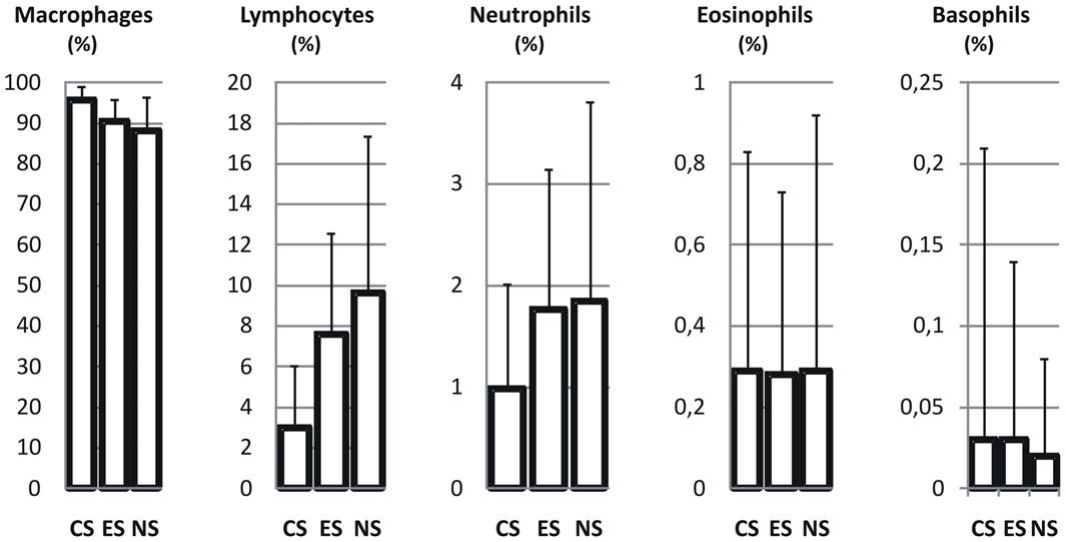
Differential cell count expressed in (% of total cells) in current smokers (CS) and neversmokers (NS) and ex-smokers (ES). Mean and standard deviation are given.

Results from comparison of data from smokers, ex-smokers and never smokers are presented in Table 4. Cell count in current smokers, exsmokers and never smokers are shown graphically in Figure 6 (cell concentrations) and in Figure 7 (percentage of cells). Both the BAL return volume and recovery were significantly lower in smokers compared to never smokers and ex-smokers. Mean cell viability was more than 90% in all three groups but was lower in smokers. Total cell number and cell concentration showed almost a four-fold increase in current smokers compared to the other two groups. This was mainly due to an increased concentration of alveolar macrophages, which was elevated by a factor five in smokers compared to both never smokers and ex-smokers. The concentration of lymphocytes was slightly higher in current smokers compared to the other groups, but the difference was not statistically significant. Measured as proportion of total cells, the percentage of lymphocytes was almost three times higher in never smokers compared to smokers and ex-smokers. The concentration of neutrophils was increased in current smokers and reached statistical significance compared to never smokers and ex-smokers. Eosinophils were rarely represented in BAL fluid, but the concentration was significantly higher in smokers than in never smokers and ex-smokers.

**Table 4:** Bronchoalveolar lavage findings in current smoking, never-smoking and ex-smoking subjects.

	Smokers		Never-smokers		Ex-smokers		Model
Variable	N	Mean (SD)	N	Mean (SD)	N	Mean (SD)	P
Return volume (mL)	128	158.4 (22.5) # §	266	179. (23.6)	41	171.7 (26.7)	<.0001
Recovery (%)	128	63.3 (9.0) # §	266	71.9 (9.4)	41	68.7 (10.7)	<.0001
Viability (%)	132	90.2 (6.0) #	292	91.7 (5.1)	42	91.6 (4.1)	0.0225
Total cell number (10^6^)	128	68.4 (34.8) # §	266	16.3 (7.6)	40	18.1 (9.5)	<.0001
Cell concentration (10^6^/L)	128	436.3 (227.2) # §	266	91.9 (41.7)	40	104.5 (48.1)	<.0001
Macrophages (%)	132	95.8 (3.3) # §	284	88.1 (8.2)	43	90.3 (5.5)	<.0001
Macrophages (10^6^/L)	128	418.9 (220.4) # §	255	80.0 (34.4)	39	92.4 (42.4)	<.0001
Lymphocytes (%)	132	2.97 (3.07) # §	284	9.66 (7.7)	43	7.60 (4.99)	<.0001
Lymphocytes (10^6^/L)	128	12.29 (12.31)	255	9.45 (14.7)	39	7.16 (5.36)	0.0528
Neutrophils (%)	131	0.98 (1.04) # §	284	1.85 (1.96)	43	1.76 (1.38)	<.0001
Neutrophils (10^6^/L)	127	3.96 (4.67) # §	255	1.63 (2.00)	39	1.95 (1.79)	<.0001
Eosinophils (%)	132	0.29 (0.54)	283	0.29 (0.63)	43	0.28 (0.45)	0.9916
Eosinophils (10^6^/L)	128	1.23 (2.71) # §	254	0.27 (0.60)	39	0.29 (0.52)	<.0001
Basophils (%)	132	0.03 (0.18)	284	0.02 (0.06)	43	0.03 (0.11)	0.8306
Basophil (10^6^/L)	128	0.09 (0.48) #	255	0.02 (0.06)	39	0.03 (0.12)	0.0340
Mast cells (per 10 visual fields)	65	2.91 (2.98)	214	2.96 (4.79)	30	2.17 (2.02)	0.6314

# Significantly different vs. Never Smokers.

§ Significantly different vs. Ex-Smokers.

### Gender differences in BAL fluid cellularity

Data from males and females separately are presented in Table 5. The smoking males were older than the smoking females (p<0.05) and had a higher cumulative cigarette consumption (p<0.05). Among smokers, both the recovered volume and the percentage of recovery were significantly lower in males compared to females. A step wise regression of pack years and age with recovery revealed that fluid recovery still was significant lower in the male smokers compared to the female smokers. In the ex-smoker group there were no significant differences between men and women.

**Table 5:** Comparison of bronchoalveolar lavage findings between current smoking, and ex-smoking subjects divided in men and women.

	Smokers			Ex-smokers		
Variable	Females	Males	p	Females	Males	p
Number	84	48		28	16	
Pack years	18.5 (2–66)	25.4 (3–84)	0.0176*	6.4 (0.2–35)	3.3 (0.2–5)	0.16
Age years mean (range)	37 (20–65)	42 (21–66)	0.0479	38 (26–54)	40 (27–54)	0.27
Return volume (mL)	163.9 (86–204)	149.2 (94–188)	0.0004***	174 (100–215)	168 (126–198)	0.46
Recovery (%)	65.6 (34–82)	59.7 (38–75)	0.0004***	70 (40–86)	67.2 (50–79)	0.45
Viability (%)	90.0 (70–100)	90.6 (79–100)	0.5736	90.9 (84–100)	92.7 (85–97)	0.16
Total Cell Number (10^6^)	68.4 (12–178)	68.3 (21–167)	0.9969	17.3 (7–61)	19 (7.0–37)	0.54
Cell concentration (10^6^/L)	420.3 (67–1143)	463.0 (164–1280)	0.3217	98 (40–285)	115 (52–207)	0.27
Macrophages (%)	95.8 (73–99)	95.8 (89–100)	0.9966	90 (73–98)	91 (83–99)	0.42
Macrophages (10^6^/L)	403.8 (64–1114)	444.1 (160–1275)	0.3364	88.2 (36–258)	99.3 (49–174)	0.41
Lymphocytes (%)	2.9 (0.5–26)	3.1 (0.5–26)	0.6867	8.1 (0.8–26)	6.7 (0.8–14)	0.37
Lymphocytes (10^6^/L)	11.3 (2–66)	14.0 (1–49)	0.2286	6.8 (1.4–17.4)	7.4 (0.7–21)	0.78
Neutrophils (%)	0.99 (0–6)	0.96 (0–6)	0.9083	1.8 (0–6.0)	1.6 (0.2–5.2)	0.69
Neutrophils (10^6^/L)	3.8 (0–30)	4.2 (0–24)	0.6078	2.0 (0–5.2)	1.9 (0.3–7.2)	0.94
Eosinophils (%)	033 (0–3.6)	0.23 (0–1.4)	0.2351	0.23 (0.0–1.4)	0.40 (0.0–1.4)	0.43
Eosinophils (10^6^/L)	0.33 (0–3.6)	0.23 (0–1.4)	0.2351	0.20 (0.0–1.3)	0.43 (0.0–2.8)	0.28
Basophils (%)	1.4 (0–15)	0.99 (0–12)	0.3902	0.007 (0.0–0.2)	0.06 (0.0–0.7)	0.29
Basophil (10^6^/L)	0.01 (0–0.2)	0.05 ( 0–2)	0.3007	0.0 (0.0–0.0)	0.07 (0.0–0.65)	0.18
Mast Cells (per 10 visual fields)	3.1 (0–13)	2.7 (0–8)	0.6397	1.5 (0.0–0.5)	3.2 (0.0–9.0	0.04

* P<0.05.

*** p<0.001.

## Discussion

In the present study, we retrospectively analyzed the effects of cigarette smoking on cellular components in BAL fluid in a large number of asymptomatic smoking volunteers. There was a reduction in the percentage of recovered fluid with increasing age. The total cell count and cell concentration were positively correlated to cumulative smoking history with considerable intra-individual variability. Compared to healthy never smokers, the cell concentration were four-fold increased with an increased concentration of all inflammatory cells, in particular macrophages.

The present study is, to our knowledge, the largest single centre investigation attempting to elucidate the effects of smoking on BAL fluid cellularity. Previous studies [4], [5], [18], [26], [27] have been performed in rather small groups and the instilled volume fluid has varied between 100–300 ml Nevertheless, it is reported a mean total cell count for asymptomatic smokers between 14.4–82.7×10^6^, a percentage of neutrophils of 0–8%, and a percentage of lymphocytes of 3–8%, which are in the same range as our results.

We performed our lavages by instilling 250 mL in the middle lobe. There are reports [4], [12], [18], [28] that larger lavage volumes contains cells representing alveoli and distal airways while smaller lavage volumes contains cells from more proximal parts of the airways. Larger volume also increases the possibility to harvest more viable cells. However, our own experience is that volumes of 300 mL or more increases the risk of lavage-related fever. In our department, with more than three decades of experience of BAL investigations, we have standardized the method by instilling 250 mL with no significant adverse effect. However, the optimal lavage volume is not yet determined.

The most striking effect of cigarette smoking is an increased number of cells, in particular macrophages [8], [14], [15], [18], [29], [30], [31] Macrophages are the first line of defence against inhaled pollutants including tobacco smoking. Macrophages obtained from smokers have a changed morphology [32], [33]. They have an altered phenotype pattern and impaired function [30], [34], [35], [36], [37], they show defect functions in killing bacteria [38] and have inhibitory effects on lymphocytes and natural killer cells [39], [40]. Fraig et al [41] found sign of respiratory bronchiolitis with increased numbers of pigmented macrophages in lung parenchyma in almost 100% of asymptomatic smokers in a biopsy material, and the intensity of inflammation was correlated to smoking history. A cigarette dose-related inflammatory response with increased numbers of macrophages and neutrophils and interleukin 1 and 6 was reported by Kuchner et al [14].

We found a lower recovery with increasing age both in smokers, and ex-smokers which have also been reported by other investigators [18], [42]. This can likely be explained by a reduced compliance in the lung parenchyma with age since smoking results in an accelerated aging process in the lungs and development of emphysema [18], [44]. In a previous paper, we demonstrated that BAL fluid recovery correlates with measures of emphysema in patients with COPD [43].

We found no differences in BAL fluid parameters between never smokers and ex-smokers. Our data indicate therefore that a normalization of BAL cells after smoking cessation in our ex-smoking group had occurred. In the study by the BAL steering committee [18] a moderate but statistically significant increase in neutrophils was observed in ex-smokers compared to never-smokers. However, the ex-smokers in that study had higher cumulative smoking history than in our study, 14.5 versus 5.3 pack years. An increased number of neutrophils in healthy ex-smokers have also been reported by other investigators [45]. The normalization process after smoking cessation is likely depending on a number of circumstances such as elapsed time since smoking cessation, duration and intensity of smoking [46], [47]. The long-time course for effects of smoking cessation on BAL cells has not been fully investigated. In a previous investigation from our department [15], we found a significant fall in total cell count one month after smoking cessation, and the values reached normal levels within six months. Rennard et al [48] investigated heavy smokers who reduced cigarette consumption from 50 to 19 cigarettes per day. There was a significant reduction in both neutrophils and macrophages but also in elastase level after two months [48]. Our study is in consistency with reversibility of smoking induced cellular changes in the lower respiratory tract in healthy ex-smokers. This is in contrast to ex-smokers with COPD and chronic bronchitis, who show signs of a persistent lower airway neutrophilic inflammation after smoking cessation [49], [50], [51] which could indicate a pathogenetic role for neutrophils in COPD.

A number of interstitial lung diseases are associated with tobacco smoking. These rare diseases are rather heterogeneous and complex with widely diverse clinical presentation. Although BAL findings alone cannot stand as diagnostic criteria in these diseases, BAL may provide valuable additional information. An increased cell concentration and a high number of pigmented macrophages in BAL is a typical feature of DIP and RB-ILD which have been exclusively reported in smokers [19], [20], [41]. In our smoking group, we found considerable intra-individual variability but increased cell concentrations, particularly regarding alveolar macrophages. There may therefore be an overlap between asymptomatic smokers and smokers with interstitial lung diseases. Thus, other diagnostic tools such as surgical lung biopsy, HRCT, physiological parameters and clinical picture have to be considered in the diagnostic work-up.

Gender differences related to BAL fluid cellularity has been investigated in few studies and there are no reports on any differences in BAL fluid cell subset related to sex [18], [26], [52]. This is in agreement with our findings. We observed, however, lower recovery of BAL fluid in smoking men compared to smoking women. This difference was still significant after correction for age and smoking history. The reason for this difference is not quite clear, but it may be explained, by the fact that males are more prone to develop emphysema than females [53]. A negative correlation between the extent of emphysema and BAL fluid recovery has previously been shown [43].

In conclusion, we are convinced that data presented in the present paper may contribute to better interpretation of BAL findings in smoking individuals, as this is the largest material on asymptomatic smokers with a well-defined smoking history.
